# Sialic Acid Utilisation and Synthesis in the Neonatal Rat Revisited

**DOI:** 10.1371/journal.pone.0008241

**Published:** 2009-12-11

**Authors:** Peter I. Duncan, Frédéric Raymond, Andreas Fuerholz, Norbert Sprenger

**Affiliations:** Nestlé Research Center, Vers-chez-les-Blanc, Lausanne, Switzerland; AgroParisTech, France

## Abstract

**Background:**

Milk is the sole source of nutrients for neonatal mammals and is generally considered to have co-evolved with the developmental needs of the suckling newborn. One evolutionary conserved constituent of milk and present on many glycoconjugates is sialic acid. The brain and colon are major sites of sialic acid display and together with the liver also of synthesis.

**Methodology/Principal Findings:**

In this study we examined in rats the relationship between the sialic acid content of milk and the uptake, utilization and synthesis of sialic acid in suckling pups. In rat milk sialic acid was found primarily as 3′sialyllactose and at highest levels between 3 and 10 days postpartum and that decreased towards weaning. In the liver of suckling pups sialic acid synthesis paralleled the increase in milk sialic acid reaching and keeping maximum activity from postnatal day 5 onwards. In the colon, gene expression profiles suggested that a switch from sialic acid uptake and catabolism towards sialic acid synthesis and utilization occurred that mirrored the change of sialic acid in milk from high to low expression. In brain sialic acid related gene expression profiles did not change to any great extent during the suckling period.

**Conclusions/Significance:**

Our results support the views that (i) when milk sialic acid levels are high, in the colon this sialic acid is catabolized to GlcNAc that in turn may be used as such or used as substrate for sialic acid synthesis and (ii) when milk sialic acid levels are low the endogenous sialic acid synthetic machinery in colon is activated.

## Introduction

Among the nine monosaccharides that are used as building blocks for mammalian glycans, sialic acid (Sia) is special. First, it is a key determinant of numerous glycan-receptor interactions involved in cell interaction and communication processes pertinent to health and disease [1]–[3]. Second, it is present in the free oligosaccharide fraction of most if not all mammalian milks [4] at levels that vary with species and time of lactation. Sia is thus amongst the first compounds ingested by the suckling newborn and this raises the question of whether or not milk Sia serves as a nutrient.

All mammals have the synthetic machinery for *de novo* synthesis of Sia, which begins with either fructose-6P and glutamine or N-acetylglucosamine and requires a series of enzymatic reactions (the biosynthesis of Sia is reviewed in [5]). The rate limiting step is catalysed by GNE (UDP-N-acetylglucosamine N-acetylmannosamine epimerase/kinase). While the synthetic machinery is present in many organs it is not clear if it is ubiquitous. In addition the absolute expression and/or activity of each gene product can vary per tissue or cell type. In adult mouse, GNE mRNA expression is highest in the liver followed by the lung and kidney [6] while Sia concentration measured in the spleen, brain and lung is considerably greater than in the liver or kidney [7]. In adult rats, considerable GNE activity has been measured in the liver, salivary gland and small intestinal mucosa while in other tissues including spleen, brain and lung the GNE activity is very low [8]. Interestingly, GNE activity in the liver of rats and guinea pigs has been reported to be low early in life during the post-natal suckling period when sialylation is considered essential for normal development and to rise significantly thereafter [9].

The brain is the major site of Sia display and has been suggested to depend on an exogenous supply during growth and development [10], [11]. Muchmore also reported that in the colon and thymus and to a lesser extent in the small intestine of the neonatal rat there is a significant albeit transient increase in Sia display in the days immediately following birth [12]. Thus as for the brain the early postnatal colon might need an exogenous supply of Sia to cope with its high needs. Such results have lead to speculation that during the neonatal suckling period *de novo* Sia production may not be sufficient to meet the needs of all tissues in the rapidly developing newborn and that Sia could serve as a conditionally essential nutrient for the suckling neonate [13].

The uptake of Sia from an exogenous, say dietary, source is thought to initiate at the plasma membrane through endocytosis. Once intracellular the material within the endocytic vesicle is delivered to the lysosome where bound Sia is removed by a resident neuraminidase and then transported out into the cytosol by the Sia transporter sialin (Slc17a5) [14]. Cytosolic Sia can then be activated through conjugation to CMP after which it is suitable to be used by Golgi-resident sialyltransferases to decorate glycoproteins and glycolipids. Alternatively, Sia was also proposed to be catabolized to N-acetylmannosamine and pyruvate [15].

Human and rodent milk is a rich source of Sia in the form of sialyloligosaccharides and thus it may be a valuable Sia supply for the newborn [4], [16], [17]. Consistent with this idea neuraminidase activity in rats and mice is highest during the suckling period and in particular in the middle and distal thirds of small intestine [18].

Dietary Sia is able to get into brain and other tissues [19], [20] however the mechanism involved and whether Sia is taken up as such or whether catabolic products reach the brain are largely unexplored. The small intestine is thought to be a major site of Sia uptake and as previously mentioned Sia has been proposed to be catabolised to ManNAc and pyruvate [15] with putative subsequent resynthesis of Sia.

Not all milk sialyloligosaccharides however, or indeed other milk oligosaccharides, are digested in the small intestine. The majority of human milk oligosaccharides seem to reach the colon where they are available for catabolism by both the microflora and host, or pass unaltered into the feces [21]–[23]. Yet their precise roles and the amount of fermented or colonic digested oligosaccharide structures are largely unknown.

Given the importance of the suckling period in the development of the newborn we sought to gain further insight into the metabolism of Sia through gene expression profiling. Our results support the view that the high concentration of Sia found in milk early during lactation favours its catabolism in the colon of the suckling pup whereas the low concentration seen at weaning stimulates the expression of genes for its synthesis and subsequent use.

## Materials and Methods

### Bioinformatics

The organism and tissue specific pattern of expression of the genes *GNE* and *Slc17a5* during development was performed with Genevestigator (http://www.genevestigator.ethz.ch), a microarray database and analysis system allowing context-driven queries [24], [25]. Additional data was obtained from the NCBI Gene Expression Omnibus (GEO) [26] and is available through GEO dataset accession number GDS1273 (http://www.ncbi.nlm.nih.gov/sites/GDSbrowser?acc=GDS1273).

### Animals and Experimental Design

Animal experiments were conducted under authorisation n° 2043 granted by the Service Vétérinaire, Etat de Vaud. Seven timed pregnant Sprague Dawley rats were obtained from Charles River Laboratories (France). The animals were individually housed, had *ad libitum* access to food and water and were maintained in a 12 hr light/dark cycle.

One day before giving birth one dam was anaesthetized with isoflurane and pups were removed by Caesarean section and dissected as described below. At birth litter sizes were adjusted to 10 pups per dam by culling or supplementation with pups from litters of the same birth date. Pups were weighed and then sacrificed by decapitation and tissues isolated. Intestinal tissue samples were rinsed with ice cold PBS to remove luminal material. The brain was separated into two hemispheres through a mid-coronal cut. All tissue samples were snap frozen in liquid N_2_ and kept frozen at −80°C before analysis.

Non-stimulated rat milk was obtained from Charles River Laboratories.

### Microarray Analysis

Brain and colon tissue samples (approximately 10 mg wet weight) were disrupted and homogenized in lysis buffer using a FastPrep instrument and lysing tubes containing ceramic beads (MP Biomedicals). Total RNA was then extracted and purified with the RNAdvance tissue kit (Agencourt) through an automated procedure on a Hamilton robotic station. After extraction, RNA was quantified with the RiboGreen RNA Quantification Kit (Molecular Probes) and monitored for quality on a Agilent 2100 Bioanalyser (RNA integrity numbers ≧8 for high quality).

All cRNA targets were synthesized, labelled and purified according to the Illumina TotalPrep RNA amplification protocol (Applied Biosystems/Ambion) using the Hamilton robotic station. Briefly, 200 ng total RNA was used to produce double-stranded cDNA followed by *in vitro* transcription and cRNA labelling with biotin. 750 ng biotin-labelled cRNA was added to the hybridization mix, which contained control oligonucleotides (such as negative and hybridization controls), hybridization buffer, and water. Then 15 µl of each hybridization mix was applied to each microarray (RatRef-12 Expression BeadChips (Illumina)). After overnight hybridization (16 hours, 58°C) the microarrays were washed to remove non-hybridized material and stained with Streptavidin-Cy3. Arrays were scanned with a BeadArray Reader (Illumina). Signal intensities were extracted and summarized in the BeadStudio software (Illumina). Data were expressed as absolute intensities. Quality control and filtering of microarray data was carried out with GeneSpring GX software (Agilent Technologies). Quality control of the data was performed on all samples with a Pearson correlation matrix and a Principal Component Analysis. One outlier (sample Colon_Embryo_Day21_rep1) was identified (data not shown) and was eliminated from the analysis. After quantile normalization, data were log_2_ transformed and analysed by ANOVA. Genes with at least one time point significantly different at a p<0.01 were selected. Genes of interest were extracted and for ease of visualization were back transformed to normalized expression levels. GraphPad Prism was used to graph figures with mean values and standard deviation.

The data have been deposited in the GEO [26] and are accessible through GEO Series accession number GSE18133 (http://www.ncbi.nlm.nih.gov/geo/query/acc.cgi?acc=GSE18133.)

### Carbohydrate Analysis

Sialyllactoses (3′ and 6′sialyllactoses) and lactose were quantified based on a previously described method [27]. Briefly, deproteinated and defatted milk, plasma and urine were subjected to high performance anion exchange chromatography (HPAEC) equipped with a CarboPac PA200 analytical column and aminotrap guard column and a pulsed amperometric detector (Dionex). Quantification was done using authentic sialyllactoses (Dextra) and lactose (Fluka).

Sia was quantified following derivatization with DMB (1,2-diamino-4,5-methylenedioxybenzene dihydrochloride) [28] and HPLC using a C18 reverse phase column (Zorbax SB-Aq Rapid Resolution column, Agilent Technologies) and fluorescence detector. Authentic N-acetylneuraminic acid (NeuAc; Dextra) and N-glycolylneuraminic acid (NeuGc; Dextra) were used as external standards.

### GNE Assay

GNE epimerase activity was assayed based on a previously described method [29]. Briefly, 30–50 mg pieces of frozen liver were homogenised in lysis buffer (10 mM Tris pH 8, 0.2% (v/v) NP-40, 1x complete protease inhibitors (Roche)) using a TissueLyser (Qiagen) and cleared by centrifugation. Total protein was measured using the Bio-Rad protein assay according the manufacturer's protocol. Supernatants were assayed for GNE activity in lysis buffer containing 50 mM sodium phosphate (pH 7.5), 10 mM MgCl_2_ and 1 mM UDP-GlcNAc (Sigma) at 37°C. Reactions were stopped by boiling and samples were subjected to HPAEC equipped with a CarboPac PA1 and PA1 guard column and a pulsed amperometric detector (Dionex). Quantification was done using authentic N-Acetyl-D-mannosamine (Sigma) as external standard. HPAEC running conditions were as follows: each run was preceded with a washing (4 min isocratic 500 mM NaOAc followed by 8 min isocratic 300 mM NaOH) and equilibration (14 min isocratic 15 mM NaOH) step. Sample running conditions were 25 min isocratic 15 mM NaOH.

## Results

### Synthesis and Uptake of Sia Seen through Public Gene Expression Data

**Figure 1:**
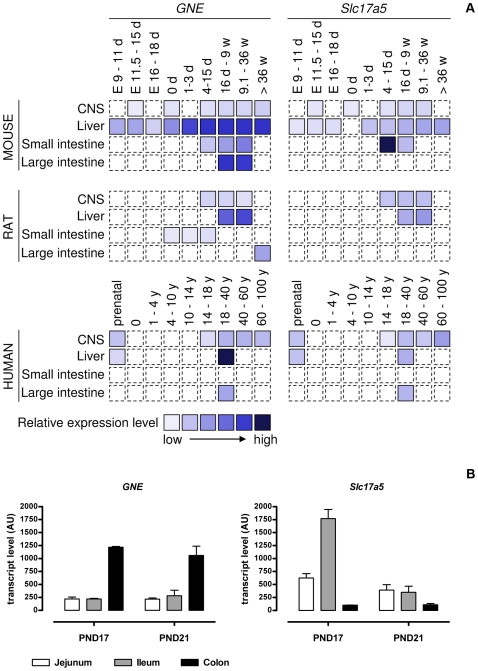
Gene expression levels of *GNE* and *Slc17a5*. A) Relative *GNE* (sialic acid synthesis) and *Slc17a5* (sialic acid uptake) expression levels in the central nervous system (CNS), liver and small and large intestine throughout development and aging in the mouse, rat and human. (E, embryonic; d, day; w, week; y, year; dashed line, not determined). B) *GNE* and *Slc17a5* expression in the intestine of 17 and 21 day old rat pups. The data was extracted from the Gene Expression Omnibus repository at http://www.ncbi.nlm.nih.gov/sites/GDSbrowser?acc=GDS1273. Mean and SD are shown; N = 3, except PND17 colon, N = 2.

Using publicly available microarray gene expression data from mouse, rat and human we monitored the RNA expression profiles of *GNE* and *Slc17a5*, genes involved in Sia synthesis and uptake respectively, over time in the brain, liver and gut (Fig. 1A). In the mouse, *GNE* expression was relatively high in both the colon and liver, while in human *GNE* expression was lower in the colon than in the liver. To complement the data available for the rat we included gene expression data for the intestine from GEO dataset GDS1273 [29] (Fig. 1B). *GNE* transcript levels were higher in colon than small intestine and high transcript levels were seen both at postnatal day 17 and 21. The most complete developmental profile of *GNE* was established for mouse liver wherein an increase was seen within the first days after birth with maximal expression occurring before weaning (usually at 21 days post-partum) (Fig. 1A). It is worth noting that while membrane fractions from brain tissue contain proportionally more Sia than membrane fractions from the liver [7] the expression of GNE there was relatively low compared to that in liver. The brain may therefore depend upon Sia synthesized elsewhere.

Gene expression data of the Sia transporter *Slc17a5* was most complete in mice where highest expression was found in the small intestine during the suckling period. A similar picture was seen in rat where highest *Slc17a5* transcript levels were expressed in the ileum at PND17 as compared to after weaning at PND21 (Fig. 1B). Although many time points are missing this suggests that maximum Sia uptake in the small intestine occurs at times when Sia is supplied via the milk to the pup. The data further suggest that highest uptake occurs in the lower small intestine. In the central nervous system no major peak of expression of *Slc17a5* was observed although the limited data available do not allow us to suggest whether or not there is increased Sia uptake into brain during the suckling period.

### Sialyllactose Levels in Rat Milk Change over Lactation

**Figure 2:**
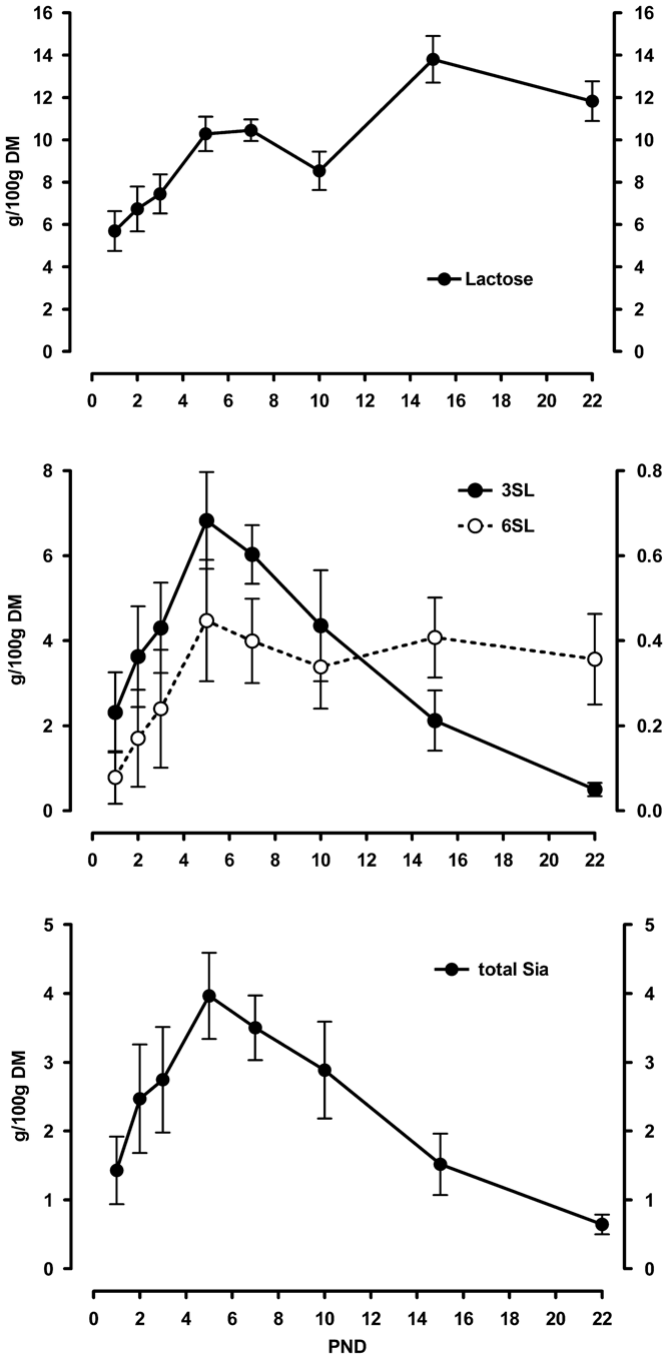
Rat milk lactose, sialyllactose and sialic acid content throughout lactation. Lactose (upper panel), the sialyllactoses (middle panel; 3SL left scale bar; 6SL right scale bar) and total sialic acid (lower panel) contents were measured in non-stimulated rat milk over time of lactation. Amounts are expressed in g per 100 g dried milk. Mean and SD are shown (N = 5).

We collected non-stimulated milk and analysed its content in lactose, sialyllactose and total Sia (Fig. 2). Lactose in milk increased throughout the first week of lactation, dropped slightly at 10 days postpartum and then increased further towards weaning. Besides the 3′- and 6′sialyllactoses no other oligosaccharides were detected. 3′Sialyllactose was the major oligosaccharide detected and was found at an ever increasing level during the first week of lactation but thereafter dropped steadily. 6′Sialyllactose was found at about ten times lower levels and also showed an increase over the first week of lactation. It then remained at a constant although low level until weaning.

The total amount of Sia in milk showed a similar profile throughout lactation to that of 3′sialyllactose and was consistent with previously published results [16], [18]. The total level increased during the first week of lactation with a peak at 5 to 7 days postpartum and then a steady decrease towards weaning. Almost all Sia present in rat milk was found to be present in the form of 3′sialyllactose and was exclusively N-acetylneuraminic acid.

With this we asked whether and how Sia related gene expression profiles change over the suckling period in colon and brain.

### Gene Expression Profiles in Colon Indicate Sia Catabolism Followed by Anabolism over Lactation

**Figure 3:**
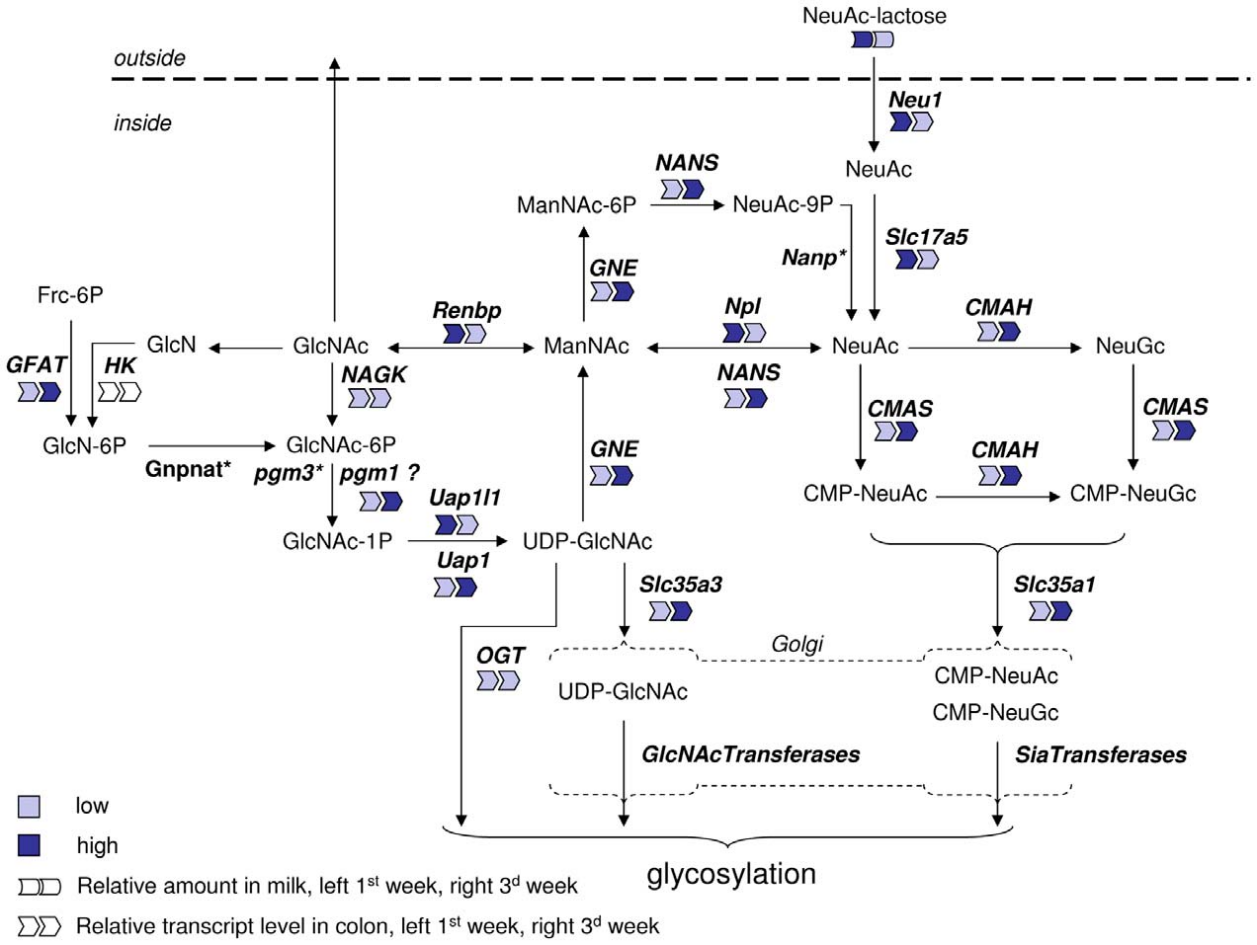
Schematic representation of sialic acid metabolism and a summary of gene expression levels in the colon. The pathways depicted are based on the KEGG aminosugar reference pathway. Full names of the indicated gene/protein are given in the text. The relative gene expression level and milk sialic acid content during first and third week of lactation are indicated by symbols and color code. (*Gene not in array or not expressed; ‘?’ no data that pgm1 can catalyse this step.)

We have depicted schematically (Fig. 3) the endogenous pathway for *de novo* Sia synthesis and possible routes for the metabolism of Sia in the newborn rat from milk-born 3′sialyllactose based on the KEGG amino sugar pathway [30] and literature data.

**Figure 4:**
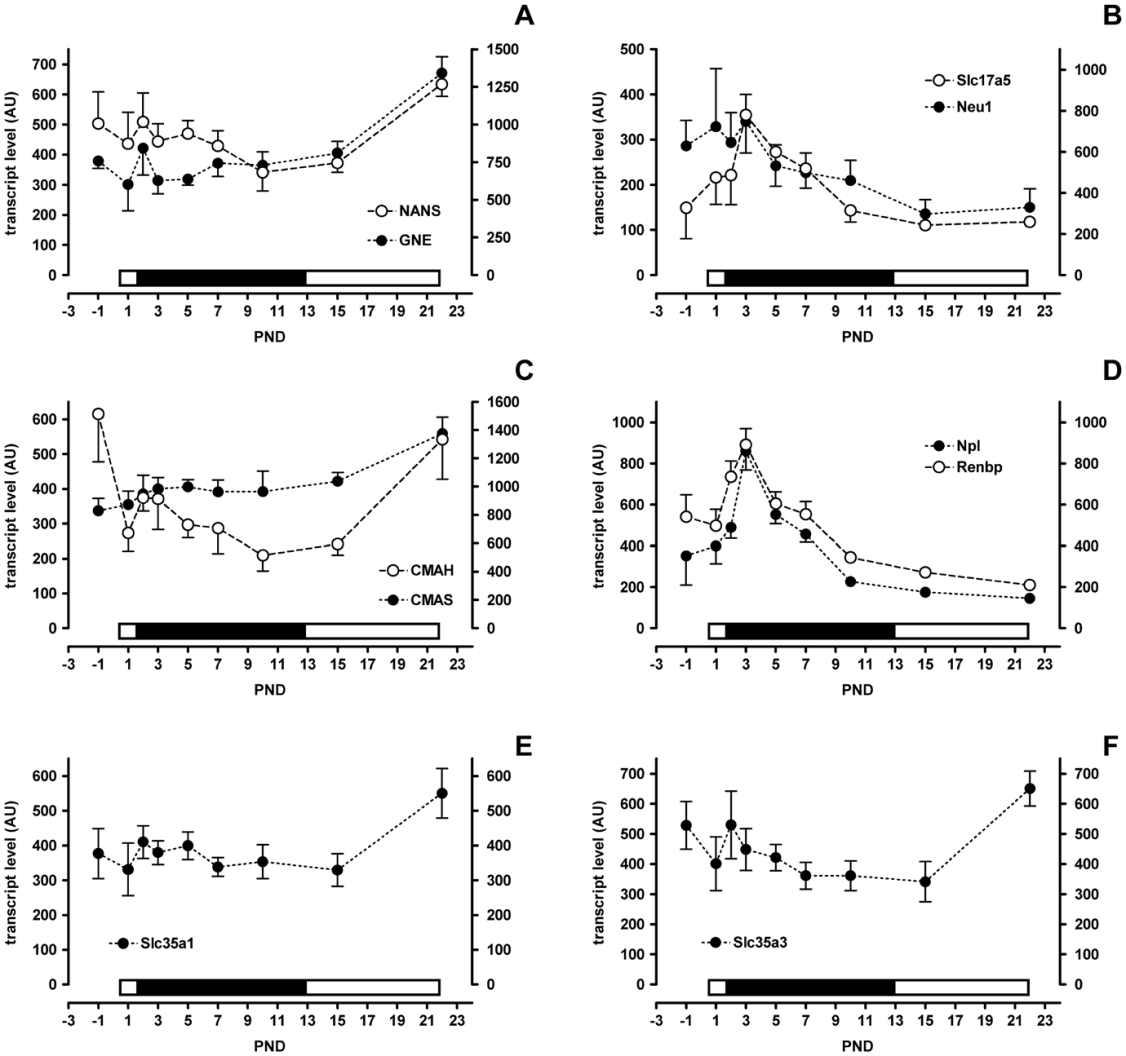
Colon gene expression levels in suckling rat pups. A–F) Filled symbols apply to the left scale bar and open symbols to the right scale bar. Mean and SD are shown (N = 6, except PND-1 N = 5). The bar above each x-axis indicates the relative level of sialic acid in milk (low, open and high, filled).

During the first half of lactation the high 3′sialyllactose in milk was accompanied by high colonic expression of neuraminidase 1 (*Neu1*) and of the Sia transporter *Slc17a5* (Fig. 4B). Together, this suggests that there is colonic cleavage and uptake of Sia during the suckling period when 3′sialyllactose delivery *via* milk is high (Figs. 3 and 4B).

In order to be added to glycan chains free Sia must be activated to CMP-Sia by the enzyme CMAS (CMP-N-acetylneuraminic acid synthase) and then be transported into the Golgi apparatus by Slc35a1 [31] (Fig. 3). The peak of expression of both *CMAS* (Fig. 4C) and *Slc35a1* (Fig. 4E) occurred at the end of lactation rather than the beginning. This suggests that the bulk of free Sia delivered *via* milk may follow another metabolic route.

We therefore next looked at the expression of genes whose products could potentially catabolise dietary derived Sia. *Npl* (N-acetylneuraminate lyase) transcript levels peaked transiently at postnatal day 3 and mirrored the expression profile of milk 3′sialyllactose (Fig. 4D). Similarly renin binding protein (*Renbp*, N-acetyl-D-glucosamine 2-epimerase) also increased to postnatal day 3 and was followed by a decrease to day 10 (Fig. 4D). Npl catalyses the breakdown of N-acetylneuraminate to N-acetyl-D-mannosamine (ManNAc) and pyruvate. Renbp epimerizes ManNAc to N-acetyl-D-glucosamine (GlcNAc) and *vice versa*.

**Figure 5:**
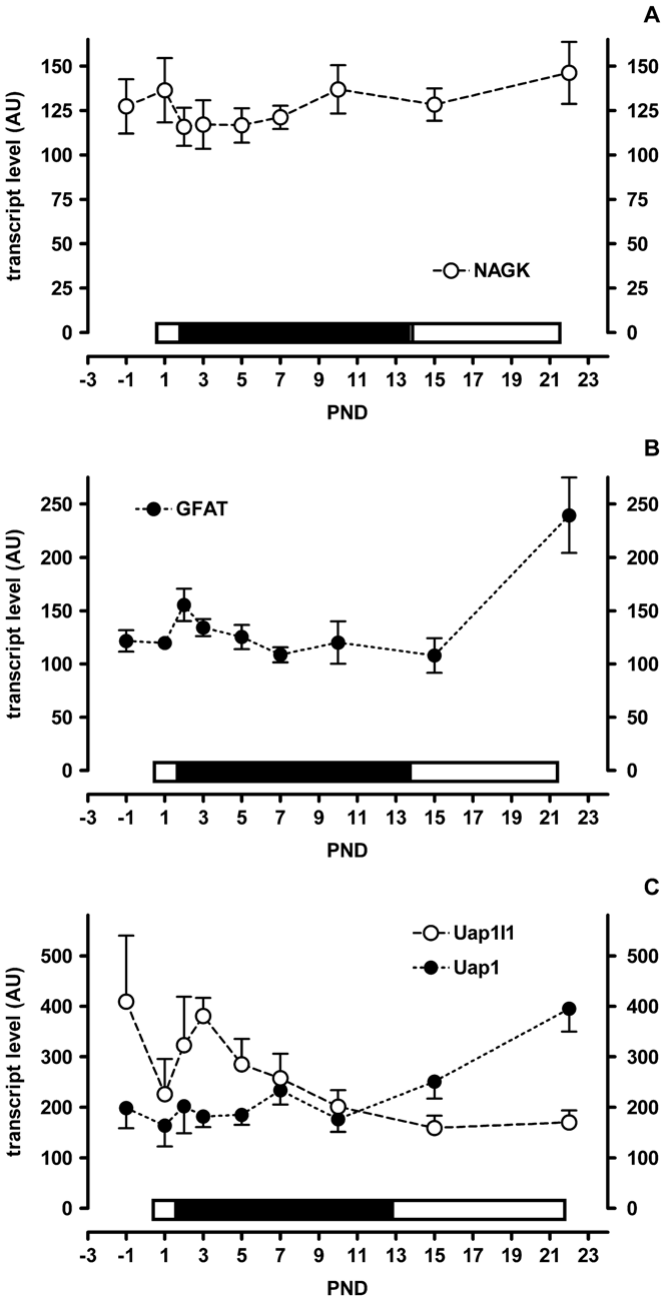
Colon gene expression levels in suckling rat pups. A–C) Filled symbols apply to the left scale bar and open symbols to the right scale bar. Mean and SD are shown (N = 6, except PND-1 N = 5). The bar above each x-axis indicates the relative level of sialic acid in milk (low, open and high, filled).

GlcNAc, the product of the sequential Npl and Renbp reactions, can then itself follow one or more metabolic fates. It can either be degraded further to glucosamine, be transported out of the cell or be used by glycosyltransferases for glycosylation reactions. Currently we do not have data to support or refute either of the first two possibilities. The biochemical pathway leading from GlcNAc to UDP-GlcNAc for use in glycosylation involves the enzymes NAGK (GlcNAc-kinase), Pgm3 (phosphoglucomutase 3) and Uap1/Uap1l1 (UDP-N-acetylglucosamine pyrophosphorylase 1/UDP-N-acetylglucosamine pyrophosphorylase 1-like 1). Subsequently, UDP-GlcNAc may be used by OGT (O-GlcNAc transferase) or transported into the Golgi apparatus by Slc35a3 for use by different glycosyltransferases. *NAGK* transcript levels did not change much during suckling (Fig. 5A). *Uap1-like1* had a transient increase shortly after birth similar to *Npl* and *Renbp*, while *Uap1* increased only later at weaning (Fig. 5C). *GFAT* (glucosamine-fructose-6phosphate-aminotransferase), the key enzyme for *de novo* GlcNAc synthesis, showed no change during early suckling but had a dramatic increase at the onset of weaning (Fig. 5B). Finally, the transporter of UDP-GlcNAc into the Golgi, *Slc35a3*, only showed a major change in expression at weaning (Fig. 4F) while that of *OGT* remained unchanged throughout (Fig. 3; expression profile not shown).

UDP-GlcNAc is also an intermediate in the *de novo* synthesis of Sia as a substrate for the rate-limiting enzyme GNE. In colon, *de novo* synthesis of UDP-GlcNAc was higher after weaning than during the suckling period as can be deduced from the gene expression profiles of *GFAT* and *Uap1* presented in figure 5. Expression of *GNE* and of the downstream enzyme *NANS* (N-acetylneuraminic acid synthase) mirrored this being higher at weaning than earlier during the suckling period (Fig. 4A).

Together, the expression profiles found in rat colon suggest that milk Sia is taken up and catabolized to N-acetylglucosamine during the early suckling period. By the end of weaning the endogenous Sia anabolic route in the colon is favoured as judged from the respective gene expression profiles.

### Plasma and Urine Sialyllactose Levels Were Highest during Times of High Sialyllactose Concentration in Milk

As milk oligosaccharides were reported to be excreted through urine we analysed plasma and urine throughout the suckling period for the presence of 3′sialyllactose, N-acetylneuraminic acid and its putative catabolic product N-acetylglucosamine.

**Figure 6:**
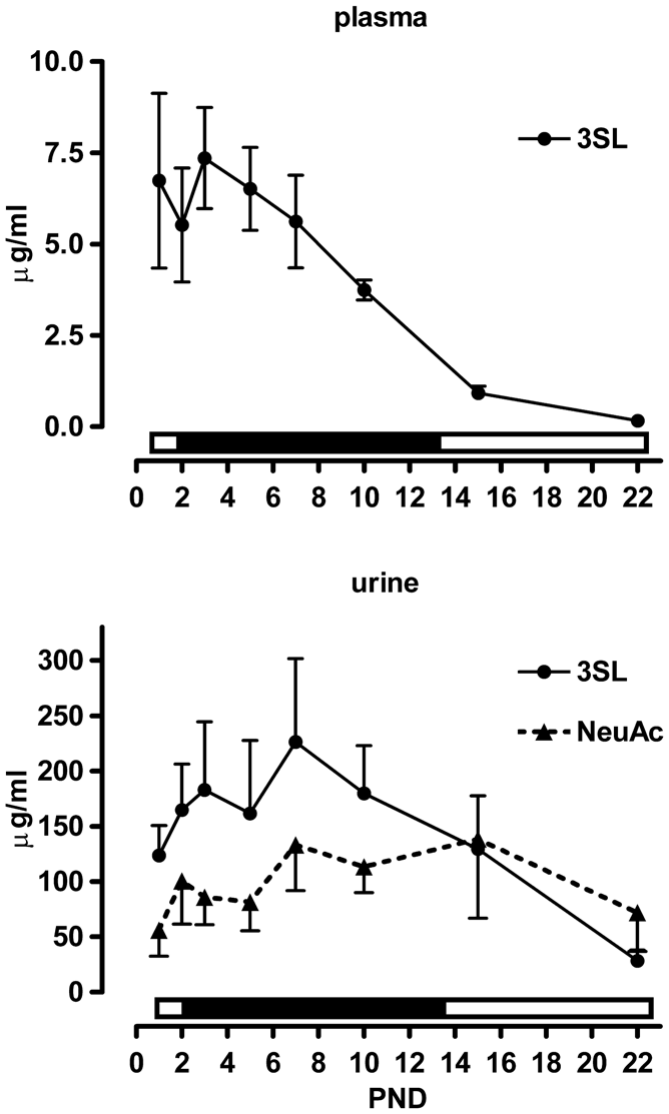
Sialyllactose and sialic acid levels in plasma and urine of suckling rat pups. A) Plasma 3′sialyllactose (3SL) and B) urine 3SL and free sialic acid (NeuAc) levels were determined in pups throughout the suckling period. Mean and SD are shown (N = 6). The bar above each x-axis indicates the relative level of sialic acid in milk (low, open and high, filled).

In plasma, we detected 3′sialyllactose, but neither free Sia nor GlcNAc (Fig. 6A). The plasma 3′sialyllactose concentration was high until PND 7 and then dropped continuously until PND 22. In urine, both 3′sialyllactose and free Sia were detected (Fig. 6B). The 3′sialyllactose content increased until PND 7 and then decreased steadily until PND 22. Free Sia showed a similar increase in the first days after birth until PND 7 but then remained constant to PND 15 and then dropped slightly by PND 22 (Fig. 6B).

### Liver Sia Synthetic Activity Increases in Parallel with Increased Sia Content in Milk

**Figure 7:**
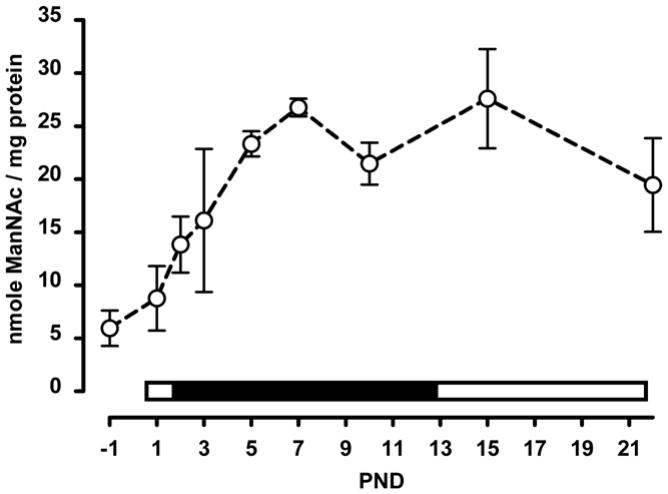
GNE activity in the livers of suckling rat pups. GNE epimerase activity was determined in liver extracts throughout the suckling period. Mean and SD are shown (N = 6). The bar above the x-axis indicates the relative level of sialic acid in milk (low, open and high, filled).

The liver is generally considered to be a major site for the synthesis Sia. This synthetic activity is reported to be low at birth and to increase towards weaning [9]. We measured the epimerase activity of GNE in the liver in suckling rats and found a steady increase in activity from prenatal day 1 to postnatal day 7. Thereafter, the GNE epimerase activity dropped slightly at 10 days and then stabilized towards weaning (Fig. 7). Interestingly, the GNE epimerase activity increased in parallel to the increasing levels of 3′sialyllactose measured in milk. This suggests that a metabolic signal may exist between the dietary Sia and liver GNE epimerase activity. This could be sialyllactose, Sia or one of its catabolic products.

### Sia Metabolism Related Gene Expression Profiles in Brain Do Not Change Much over Time

**Figure 8:**
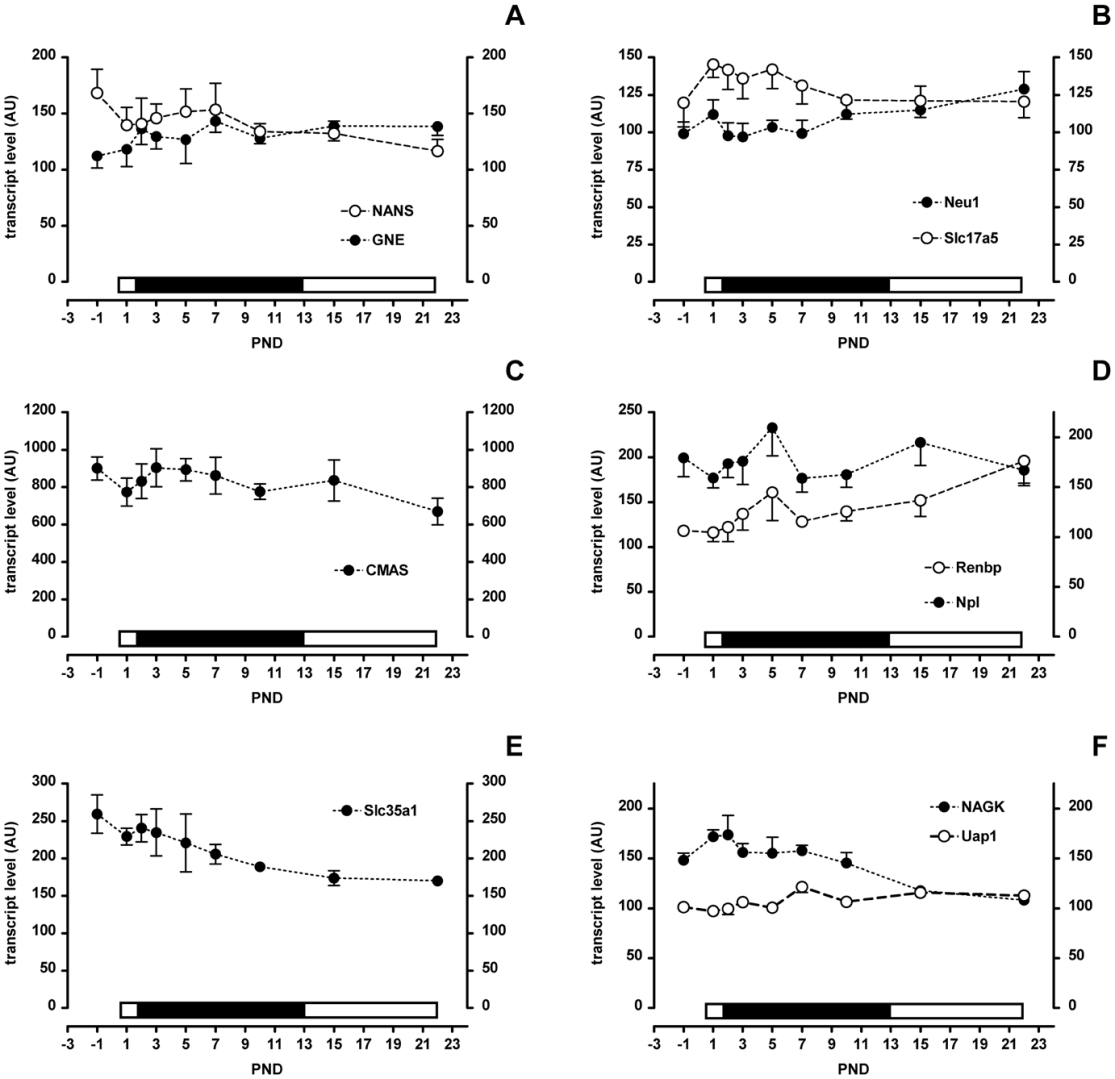
Brain gene expression levels in suckling rat pups. A–F) Filled symbols apply to the left scale bar and open symbols to the right scale bar. Mean and SD are shown (N = 6). The bar above each x-axis indicates the relative level of sialic acid in milk (low, open and high, filled).

The brain has been suggested to use an exogenous supply of Sia for development and growth [10], [11]. To determine how the brain reacts to ingested milk sialyllactose we measured gene expression levels throughout the suckling period. The endogenous Sia synthetic route with *GNE*, *NANS* and *CMAS* showed a relatively constant expression profile from birth to the end of weaning (Fig. 8A & C). The CMP-Sia transporter *Slc35a1* showed a low but constant reduction in expression throughout (Fig. 8E) which suggests that less Sia incorporation in glycoconjugates occurs as the pups are weaned. As for genes potentially involved in an exogenous supply of Sia to the brain expression of *Neu1* was relatively constant while that of the Sia transporter *Slc17A5* was marginally higher from PND 2 to 7 (Fig. 8B). *Npl* and *Renbp* both had a peak of expression at PND 5 and whereas *Npl* expression was stable or declining by weaning that of *Renbp* was increasing (Fig. 8D). Finally in the metabolism of GlcNAc *NAGK* had a peak of expression in the first two days following birth and then declined slowly to weaning. Expression of *Uap1* on the other hand was constant throughout the suckling period (Fig. 8F).

The slight changes in gene expression do neither favour the endogenous biosynthetic pathway nor an exogenous supply as a primary source of Sia during early brain growth and development.

## Discussion

Given the importance of the suckling period in the development of the newborn rat we sought to gain further insight into the synthesis and metabolism of Sia. To achieve this we compared throughout the suckling period the concentration of Sia in milk with (i) the expression profiles of genes involved in Sia metabolism in the colon and brain, major sites of synthesis and incorporation of Sia, and (ii) the Sia synthetic capacity in the liver, also a major site of synthesis. It is worth emphasizing that the results of gene expression profiling do not necessarily correlate with the corresponding gene product levels, their catalytic activities or the flux of their substrates and products. Gene expression results do however provide important data that can be used to direct subsequent research.

Early in lactation Sia levels, principally as 3′sialyllactose, were highest and in the colon this correlated with the expression profile of catabolic genes. This suggests that Sia can be cleaved from sialyl-glycoconjugates, absorbed and subsequently catabolised to yield ManNAc, pyruvate and subsequently GlcNAc. These findings support the notion that milk Sia is a nutrient for the suckling neonate. At the end of weaning, when Sia levels in milk were lowest, colonic gene expression had switched such that those involved in *de novo* UDP-GlcNAc and CMP-Sia synthesis together with their respective Golgi resident transporters showed the highest relative expression. Thus, in the colon as the dietary source of Sia diminishes the body may compensate by up regulating Sia synthetic genes.

Several lines of evidence have lead others to suggest that milk Sia can be a nutrient for the neonate early during the suckling period. Over 3 decades ago a correlation was described between the neuraminidase activity in the middle and distal small intestine and the milk Sia content that led the authors to suggest that milk Sia is liberated and used by the suckling newborn [18]. In accordance, high Sia uptake is suggested from the high transient expression of *Slc17a5* in the distal small intestine and of *Neu1* and *Slc17a5* in the colon. Interestingly beyond uptake genes we also saw *Npl* and *Renbp* upregulated during the period when the concentration of Sia in milk was at its peak. While these two genes are part of the same biochemical pathway the direction of reaction is unclear (Fig. 3). Together Npl and Renbp may drive an alternative Sia synthetic route that is active when GNE expression is low or a catabolic route during times when Sia delivery is high. The former is possible but unlikely because GNE knockout mice are embryo lethal and the GNE deficient cells that were tested cannot make Sia from GlcNAc indicating that no salvage pathway exists [32]. It is thus more likely that Npl and Renbp catalyse the catabolism of Sia to GlcNAc. Indeed, Npl was reported to be mainly involved in Sia catabolism *in vivo* [33], and Renbp is thought to epimerize ManNAc to GlcNAc rather then the reverse [34]. It would be interesting to determine if the same expression profiles of *Npl* and *Renbp* occur in the small intestine. Finally results from tracer feeding experiments support the view that nutritionally supplied Sia (as sialyllactose or free Sia) can be a nutrient and preferentially early during the suckling period [15], [19], [20], [35], [36]. In 3 day old rat pups approximately 30% of administered radioactivity remained in the body after 6 hr while 70% was excreted via the lungs (i.e. as CO2), kidney and feces [20]. In 20 day old mice only approximately 1.5% of the administered radioactivity was retained in the body after 6 hr [19]. Morgan and Winick also showed that newborn rats incorporate considerably more tracer early during the suckling period than at the end of weaning [37]. While the nature of the labelled molecule(s) present in the tissues was not identified in these studies Nöhle and Schauer proposed a model where Sia is cleaved from a glycoconjugate followed by Npl catalysed cleavage to ManNAc and pyruvate [19]. Our results support this model and suggest that ManNAc can be further epimerized by Renbp to GlcNAc.

The brain incorporates a large amount of Sia during development which begins *in utero* but continues postnatally (data not shown; [38], [39]). Postnatally the sources and their relative contributions of Sia for use in the brain (e.g. synthesized within the brain, in another tissue (e.g. liver) or from the diet) are currently unknown. The Sia transporter Slc17a5 has been reported to be expressed throughout the adult rodent brain and during embryonic development highest levels are seen in the central and peripheral nervous system [40]. Also radioactivity from radiolabelled Sia (either as free sugar or as sialyllactose) administered to suckling animals can reach the brain although the biochemical pathway followed is not known [19], [20], [36]. Such results have led to the suggestion that the brain gets at least some of its Sia from exogenous sources [10], [11]. Our gene expression results in the neonatal brain do not provide additional evidence for a significant use of exogenous Sia during this time. Studying the brain as a whole as was done here may have masked important regional differences where significant use of exogenously supplied Sia occurs.

The liver may be a source of Sia for other tissues and organs. In the liver the epimerase activity of GNE, the rate limiting enzyme of Sia synthesis, increased during the first week of lactation in parallel to the milk Sia concentration. Whether milk Sia is the stimulus for this activity increase is unknown however Wang *et al*. have previously shown that piglets provided a diet enriched in Sia increased expression of *GNE* mRNA in both the liver and hippocampus [41]. At present we do not know if rat liver *GNE* gene expression parallels that of its enzymatic activity during early development.

Ingested milk oligosaccharides are also excreted unaltered and in significant amounts in the urine and feces [19], [21]–[23]. In human infants about 0.5 to 1% of ingested milk oligosaccharides were reported to be excreted in urine [23], [42]. In rats we also found part of the ingested sialyllactose in urine and estimated that maximally 1% of ingested sialyllactose was excreted into urine (data not shown).

Altogether these results prompt the following questions. (i) What is the biological role of the bulk of ingested sialyllactose that is not digested and (ii) what is the selective advantage of delivering Sia to the suckling pup instead of ManNAc or GlcNAc? A possible answer to both questions is their decoy function. Indeed there is a longstanding hypothesis that milk oligosaccharides serve a decoy function to prevent potentially dangerous microbes from doing harm to the immunologically fragile neonate by reducing microbe-host interactions [43]. Along the same lines free milk oligosaccharides such as sialyllactoses may modulate interactions directly within the host. This could include interactions between cells, between receptor and ligands and between receptors and co-receptors. Such modulation is to date largely unexplored. Further, systemically circulating sialyllactose may serve as a source of Sia for extra-intestinal tissues such as the brain as suggested above.

There is thus good evidence that milk Sia is digested in the neonatal gut and used as a nutrient and that it may be partially catabolized to pyruvate, ManNAc and GlcNAc. To our knowledge it is not known to what extent or where these catabolic products are reused for Sia synthesis, used as such or further catabolized. A detailed understanding of the metabolic fate of dietary Sia is needed to answer these questions and could provide insight into the possible evolutionary advantages of expressing high levels of Sia rather than ManNAc or GlcNAc in milk.
